# Association of Academic Medical Center Presence With Clinical Outcomes at Neighboring Community Hospitals Among Medicare Beneficiaries

**DOI:** 10.1001/jamanetworkopen.2022.54559

**Published:** 2023-02-01

**Authors:** Laura G. Burke, Ryan C. Burke, E. John Orav, Ciara E. Duggan, Jose F. Figueroa, Ashish K. Jha

**Affiliations:** 1 Department of Emergency Medicine, Beth Israel Deaconess Medical Center, Boston, Massachusetts; 2 Department of Health Policy and Management, Harvard T.H. Chan School of Public Health, Boston, Massachusetts; 3 Department of Emergency Medicine, Harvard Medical School, Boston, Massachusetts; 4 Division of General Internal Medicine, Department of Medicine, Brigham and Women’s Hospital, Boston, Massachusetts; 5 Brown University School of Public Health, Providence, Rhode Island

## Abstract

**Importance:**

Studies suggest that academic medical centers (AMCs) have better outcomes than nonteaching hospitals. However, whether AMCs have spillover benefits for patients treated at neighboring community hospitals is unknown.

**Objective:**

To examine whether market-level AMC presence is associated with outcomes for patients treated at nonteaching hospitals within the same markets.

**Design, Setting, and Participants:**

This retrospective, population-based cohort study assessed traditional Medicare beneficiaries aged 65 years and older discharged from US acute care hospitals between 2015 and 2017 (100% sample). Data were analyzed from August 2021 to December 2022.

**Exposures:**

The primary exposure was market-level AMC presence. Health care markets (ie, hospital referral regions) were categorized by AMC presence (percentage of hospitalizations at AMCs) as follows: no presence (0%), low presence (>0% to 20%), moderate presence (>20% to 35%), and high presence (>35%).

**Main Outcomes and Measures:**

The primary outcomes were 30-day and 90-day mortality and healthy days at home (HDAH), a composite outcome reflecting mortality and time spent in facility-based health care settings.

**Results:**

There were 22 509 824 total hospitalizations, with 18 865 229 (83.8%) at non-AMCs. The median (IQR) age of patients was 78 (71-85) years, and 12 568 230 hospitalizations (55.8%) were among women. Of 306 hospital referral regions, 191 (62.4%) had no AMCs, 61 (19.9%) had 1 AMC, and 55 (17.6%) had 2 or more AMCs. Markets characteristics differed significantly by category of AMC presence, including mean population, median income, proportion of White residents, and physicians per population. Compared with markets with no AMC presence, receiving care at a non-AMC in a market with greater AMC presence was associated with lower 30-day mortality (9.5% vs 10.1%; absolute difference, −0.7%; 95% CI, −1.0% to −0.4%; *P* < .001) and 90-day mortality (16.1% vs 16.9%; absolute difference, −0.8%; 95% CI, −1.2% to −0.4%; *P* < .001) and more HDAH at 30 days (16.49 vs 16.12 HDAH; absolute difference, 0.38 HDAH; 95% CI, 0.11 to 0.64 HDAH; *P* = .005) and 90 days (61.08 vs 59.83 HDAH; absolute difference, 1.25 HDAH; 95% CI, 0.58 to 1.92 HDAH; *P* < .001), after adjustment. There was no association between market-level AMC presence and mortality for patients treated at AMCs themselves.

**Conclusions and Relevance:**

AMCs may have spillover effects on outcomes for patients treated at non-AMCs, suggesting that they have a broader impact than is traditionally recognized. These associations are greatest in markets with the highest AMC presence and persist to 90 days.

## Key Points

**Question:**

Is the presence of an academic medical center (AMC) within a health care market associated with better outcomes for patients treated at neighboring nonteaching hospitals?

**Findings:**

In this retrospective cohort study of 22 509 824 hospitalizations among older Medicare beneficiaries at US acute care hospitals from 2015 to 2017, receiving care in a market with high AMC presence was associated with lower mortality and a greater number of healthy days at home among patients treated at non-AMCs.

**Meaning:**

These findings suggest that AMCs may have spillover effects on outcomes for patients treated at neighboring nonteaching hospitals.

## Teaser

This cohort study examines whether market-level academic medical center presence is associated with outcomes for Medicare patients treated at nonteaching hospitals within the same markets.

## Introduction

Variation in quality among clinicians and hospitals in the US is well documented, with meaningful differences in serious outcomes such as mortality.^1,2^ Such differences in health care quality result from multiple factors, including characteristics of clinicians (eg, age, gender, and patient volume),^3,4,5,6,7^ hospitals (eg, volume and complexity),^8,9,10^ and the health care market and community.^11,12^ One factor that has been identified as being associated with overall quality is hospital teaching status. Multiple studies^13,14,15,16,17,18,19,20,21^ have demonstrated better outcomes, on average, for patients treated at academic medical centers (AMCs) compared with those treated at nonteaching hospitals.

However, given that AMCs account for a minority of US hospitals (approximately 6%),^17^ it is not feasible for most patients to receive treatment at an AMC. However, it is possible that AMCs provide local benefits beyond direct patient care. Formal and informal arrangements between AMCs and community hospitals may include sharing clinical staff, disseminating protocols, and serving as a source of newly trained physicians. In addition, patients in communities with greater AMC presence may have better access to clinical trials or other advances. Yet, evidence on possible spillover benefits of AMCs is lacking. A fuller understanding of the broader impact of AMCs is especially important given the substantial federal spending on graduate medical education.^22,23^

We sought to address this evidence gap using national Medicare claims to investigate the following questions: First, how does AMC presence vary among health care markets in the US? Second, do patients hospitalized at non-AMCs have lower mortality when a health care market has greater AMC presence? Third, do patients hospitalized at non-AMCs have a greater number of healthy days at home (HDAH), a population-based outcome measure that combines mortality and total time spent out of facility-based health care settings, when hospitalized in a market with greater AMC presence?

## Methods

### Data

#### Study Sample

The Office of Human Research Administration at the Harvard T.H. Chan School of Public Health approved this cohort study. Informed consent could not be practically obtained for this retrospective study. This study follows the Strengthening the Reporting of Observational Studies in Epidemiology (STROBE) reporting guideline for observational studies.

We identified all inpatient discharges among traditional Medicare beneficiaries aged 65 years and older between 2015 and 2017 (100% inpatient file). We classified principal discharge diagnosis into Healthcare Utilization Project Clinical Classification Software categories. We identified beneficiary characteristics (age, sex, Medicaid eligibility, race, and chronic conditions obtained from the Chronic Conditions Warehouse file) and whether the hospitalization ended in interhospital transfer. Given the known racial disparities in health care access and quality, beneficiary race was identified and reported as defined in Medicare data, which is primarily derived from Social Security Administration data.^24^

#### Health Care Markets

We defined health care markets by the Dartmouth Atlas hospital referral regions (HRRs), which represent regional markets for tertiary medical care. Using the Dartmouth Atlas, the Centers for Medicare & Medicaid Services Public Use files, and the American Community Survey (2011-2015), we identified the following market characteristics: total population, median income, percentage of residents with income below the poverty line, percentages of Black residents and White residents, number of physicians per 100 000 population, number of primary care physicians per 100 000 population, and number of hospital full-time equivalents (FTEs) per 1000 population.

#### Hospitals

We identified acute care hospitals in all 50 states and the District of Columbia from the American Hospital Association Annual Survey. We defined hospital teaching status by intern or resident-to-bed ratio (IRB) from the Medicare cost reports. We classified AMCs as those with an IRB of 0.25 or greater and non-AMCs as those with an IRB less than 0.25.^21,25^

### Outcomes

Our primary outcome was mortality (from the Master Beneficiary Summary File) within 30 days of an inpatient stay. To determine whether any associations persisted, we also identified 90-day mortality. In addition, we examined HDAH, an outcome developed by the Medicare Payment Advisory Commission described in prior studies.^26,27,28,29^ To calculate 30-day HDAH, we subtracted from 30 the number of days in the follow-up period in which the beneficiary was deceased or in the following settings: inpatient, observation, skilled nursing facility, outpatient emergency department, inpatient psychiatry, inpatient rehabilitation, and long-term hospital.

### Statistical Analysis

Data were analyzed from August 2021 to December 2022. For each HRR, we calculated the number of AMCs in 2015, as well as the proportion of hospitalizations managed at AMCs in 2015 to 2017. We created the 4 market categories: no AMC presence (0% of hospitalizations managed at AMCs), low (>0% to 20%), moderate (>20% to 35%),and high (>35%) AMC presence.

One concern about comparing these groups is that they have demographic and health system differences beyond AMC presence that are associated with differential health outcomes. We, therefore, examined how key characteristics varied among the 4 market groups and tested for differences using analysis of variance for means and Kruskal-Wallis for medians.

After calculating raw mortality rates, we constructed a set of linear probability models with 30-day mortality as the outcome and market-level AMC presence as the categorical variable. Our initial model adjusted for year, principal discharge diagnosis, geographic region (Northeast, Midwest, South, and West), and hospital random effects. Given that patient characteristics vary widely and are associated with patient outcomes,^30,31^ our subsequent model further incorporated the diagnosis-related group weight as an indicator of the complexity of the acute condition as well as patient demographics (age, sex, and Medicaid eligibility) and chronic conditions. To account for nonrandom distribution of AMCs and hypothesized differences in the types of communities where AMCs are located, our main model further incorporated market characteristics (total population, proportion of Black residents, median income, physicians per 100 000 population, and hospital FTEs per 1000 population). We calculated mean adjusted rates of 30-day mortality for the 4 market groups and repeated these models for the outcome of 90-day mortality. We specified analogous sets of linear regression models with the outcomes of 30-day and 90-day HDAH. SAS code for the main models is presented in eTable 1 in Supplement 1.

In secondary analyses, to evaluate the association for a nonlinear trend, we created a locally estimated scatterplot smoothing curve, plotting the proportion of hospitalizations in an HRR managed at an AMC on the x-axis and unadjusted 30-day mortality on the y-axis. We repeated our analysis for adjusted 30-day mortality using market AMC presence as a continuous variable, adjusting for patient and market characteristics.

We considered the possibility that any association between market-level AMC presence and patient outcomes at non-AMCs could be associated with unobserved between-market differences in patient severity, rather than spillover effects of AMCs. If that were the case, we might expect to see similar patterns of outcomes for patients treated at AMCs themselves within these same market categories. Thus, we repeated our models for mortality among patients hospitalized at AMCs, with AMC presence as the categorical variable and adjusting for the same set of covariates.

One potential mechanism for the association between AMC presence and mortality for patients at non-AMCs could be the differential ability to transfer seriously ill patients. Timely transfer is associated with better outcomes for key conditions.^32^ It is, thus, possible that equivalently ill patients in markets with low AMC presence may have worse outcomes if they lack access to tertiary care transfer. We, therefore, repeated our models for 30-day and 90-day mortality, incorporating market-level transfer rate. We conducted analyses using SAS statistical software version 9.4 (SAS Institute), using a significance level of *P* < .05 and calculating 2-sided 95% CIs.

## Results

Our sample include 22 509 824 total hospitalizations from 2015 to 2017, of which 18 865 229 (83.8%) were at non-AMCs. The median (IQR) age of patients was 78 (71-85) years, and 12 650 521 hospitalizations (56.2%) were among women. Hospitalizations missing an HRR designation (169 768 hospitalizations [0.8%]) were excluded from the analysis.

### Market-Level Variation in AMC Presence

**Figure 1.**
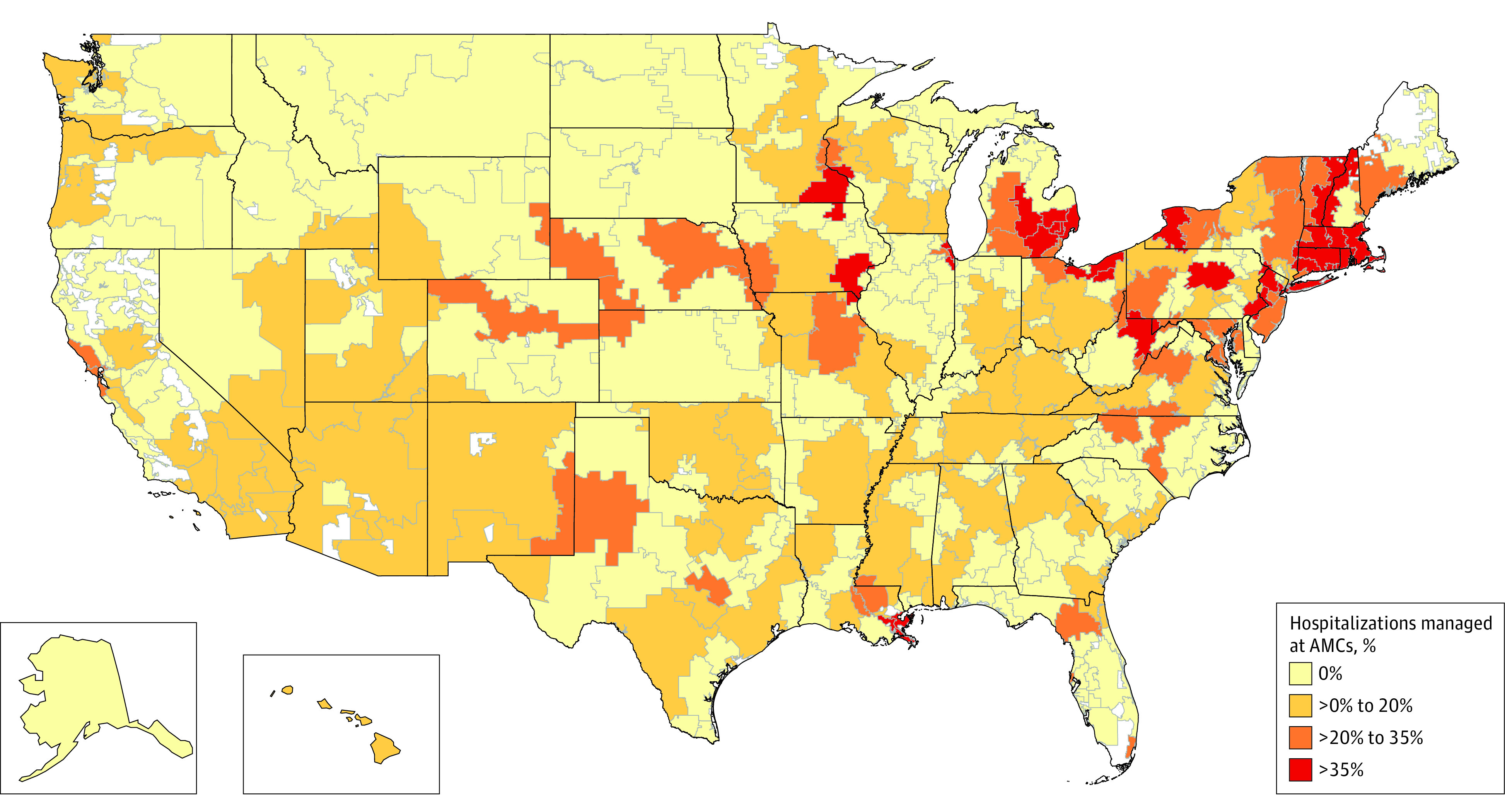
Variation Among Health Care Markets With Respect to the Proportion of Hospitalizations Managed at Academic Medical Centers (AMCs) Health care markets are defined by Dartmouth Atlas hospital referral regions.

The mean number of AMCs per HRR was 0.93 (range, 0-24 AMCs). Of the 306 HRRs in the US, 191 (62.4%) had no AMCs in 2015. Sixty-one (19.9%) had 1 AMC, 24 (7.8%) had 2 AMCs, 12 (3.9%) had 3 AMCs, and 18 (5.9%) had 4 or more AMCs (eFigure 1 in Supplement 1). Accordingly, the median HRR had 0 hospitalizations managed at AMCs, the mean was 10.6%, the 75th percentile was 14.3%, and the 95th percentile was 54.9% (eFigure 2 in Supplement 1). Figure 1 shows the geographic distribution of markets with no (178 markets), low (67 markets), moderate (31 markets), and high (30 markets) AMC presence.

### Characteristics of Markets and Hospitalizations

The demographic characteristics of the overall population residing in the 4 market categories and the health system characteristics are presented in Table 1; the characteristics of hospitalized Medicare beneficiaries in our study sample are shown in Table 2. There were differences among the 4 groups for the following characteristics: median income and mean total population, proportion of White residents, poverty rate, and physicians, nurses, and hospital FTEs per capita (Table 1). Markets with no AMC presence had the lowest median (IQR) income ($48 609 [$43 969-$55 148]) as well as mean (SD) population (532 507 [480 409] individuals), mean (SD) physicians per 1000 population (187.0 [26.7] physicians), and the highest mean poverty rate (16.4%; 95% CI, 15.7%-17.0%) and proportion of White residents (81.1%; 95% CI, 79.4%-82.9%) (Table 1). The characteristics of Medicare beneficiaries hospitalized at non-AMCs were similar across the 4 groups (Table 2).

**Table 1.** Key Market Demographic and Health System Characteristics by Degree of AMC Presence^a^

Characteristics	AMC market presence				*P* value^b^
	None (n = 178 HRRs)	Low (n = 67 HRRs)	Moderate (n = 31 HRRs)	High (n = 30 HRRs)	
Demographic characteristics					
Population, mean (SD), individuals	532 507 (480 409)	1 995 491 (1 647 577)	1 330 008 (864 682)	1 526 098 (1 406 807)	<.001
Black population, mean (95% CI), %	9.8 (8.2-11.4)	11.7 (9.0-14.4)	11.2 (7.9-14.5)	14.7 (10.1-19.4)	.13
White population, mean (95% CI), %	81.1 (79.4-82.9)	74.6 (71.3-77.8)	77.2 (72.8-81.5)	73.3 (66.7-79.8)	.001
Poverty rate, mean (95% CI), %	16.4 (15.7-17.0)	16.3 (15.4-17.2)	14.2 (12.8-15.6)	15.1 (13.1-17.0)	.04
HRR income, median (IQR), $	48 609 (43 969-55 148)	53 289 (46 984-61 177)	54 627 (49 310-70 589)	58 840 (49 175-69 354)	<.001
Health system characteristics, mean (SD), per 100 000 population					
Physicians	187.0 (26.7)	199.1 (27.4)	217.3 (31.7)	234.0 (29.2)	<.001
Primary care practitioners	69.0 (11.3)	70.9 (11.5)	78.2 (12.8)	82.9 (10.4)	<.001
Nurses	4.2 (0.8)	4.1 (0.7)	4.2 (0.7)	4.7 (0.8)	.005
Hospital beds	2.2 (0.6)	2.1 (0.5)	2.0 (0.4)	2.0 (0.4)	.02
Hospital full-time equivalents	15.4 (3.6)	14.1 (2.6)	15.3 (2.6)	17.3 (2.7)	<.001

Abbreviations: AMC, Academic Medical Center; HRR, hospital referral region.

^a^ Table was created using data from the Dartmouth Atlas, the Centers for Medicare & Medicaid Services Public Use Files, and the American Community Survey (2011-2015).

^b^ Analysis of variance was used to formally test for differences in mean market characteristics across the 4 groups and Kruskal-Wallis for differences in median income.

**Table 2.** Characteristics of Patients Hospitalized at Non-AMCs, Stratified by Market-Level AMC Presence^a^

Patient characteristics	Patients, No. (%), by AMC presence			
	None	Low	Moderate	High
Total sample size	7 542 967 (40.0)	7 415 020 (39.3)	2 226 995 (11.8)	1 555 818 (8.3)
Transfer out	158 789 (2.1)	154 072 (2.1)	64 011 (2.9)	48 420 (3.11)
Age, median (IQR), y	78 (71-85)	78 (71-85)	79 (72-86)	79 (72-86)
Age category, y				
65-74	2 838 681 (37.6)	2 855 221 (38.5)	787 341 (35.4)	522 754 (33.6)
75-84	2 744 557 (36.4)	2 656 589 (35.8)	780 480 (35.1)	542 496 (34.9)
≥85	1 959 729 (26.0)	1 903 210 (25.7)	659 174 (29.6)	490 568 (31.5)
Sex				
Male	3 354 344 (44.5)	3 234 464 (43.6)	952 374 (42.8)	663 670 (42.7)
Female	4 188 623 (55.5)	4 180 556 (56.4)	1 274 621 (57.2)	892 148 (57.3)
Race and ethnicity^b^				
Asian	47 557 (0.6)	147 603 (2.0)	27 021 (1.2)	13 117 (0.8)
Black	577 188 (7.7)	607 859 (8.2)	203 240 (9.1)	117733 (7.6)
Hispanic	94 490 (1.3)	125 077 (1.7)	46 937 (2.1)	14 006 (0.9)
North American Native	49 374 (0.7)	56 629 (0.8)	4899 (0.2)	1201 (0.1)
White	6 674 752 (88.5)	6 327 034 (85.3)	1 903 290 (85.5)	1 380 306 (88.7)
Other^c^	56 679 (0.8)	102 255 (1.4)	23 944 (1.1)	16 242 (1.0)
Unknown	42 927 (0.6)	48 563 (0.7)	17 664 (0.8)	13 213 (0.9)
Medicaid eligible				
Yes	1 623 713 (21.5)	1 687 779 (22.8)	513 914 (23.1)	349 986 (22.5)
No	5 919 179 (78.5)	5 727 156 (77.2)	1 713 044 (76.9)	1 205 804 (77.5)
Missing	75 (0.0)	85 (0.0)	37 (0.0)	28 (0.0)
Principal discharge diagnosis^d^				
Septicemia	646 696 (8.6)	682 518 (9.2)	186 473 (8.4)	135 174 (8.7)
Osteoarthritis	448 151 (6.0)	431 212 (5.8)	128 718 (5.8)	84 876 (5.5)
Pneumonia	305 871 (4.1)	305 645 (4.1)	89 735 (4.1)	61 319 (4.0)
Dysrhythmia	299 056 (4.0)	272 420 (3.7)	80 745 (3.6)	60 536 (3.9)
Congestive heart failure	293 412 (3.9)	282 981 (3.8)	90 210 (4.1)	66 162 (4.3)
Missing	30 325 (0.4)	29 164 (0.4)	8605 (0.4)	5963 (0.4)
Chronic conditions				
Congestive heart failure	3 679 111 (48.8)	3 698 995 (49.9)	1 108 594 (49.8)	805 579 (51.8)
Chronic kidney disease	4 424 445 (58.7)	4 429 569 (59.7)	1 307 536 (58.7)	919 151 (59.1)
Chronic obstructive pulmonary disease	2 960 746 (39.3)	2 829 882 (38.2)	868 897 (39.0)	612 216 (39.4)
Depression	2 914 079 (38.6)	2 910 393 (39.3)	901 661 (40.5)	636 077 (40.9)
Acute myocardial infarction	528 719 (7.0)	507 219 (6.8)	149 961 (6.7)	111 174 (7.2)

Abbreviation: AMC, Academic Medical Center.

^a^ Table was created using data on hospitalizations of Medicare beneficiaries (100% inpatient file) at non-AMCs from 2015-2017 and data from the Dartmouth Atlas.

^b^ Race and ethnicity categories were defined in the Medicare data set.

^c^ Other is defined in the Medicare Beneficiary Summary File as a separate category.

^d^ Principal discharge diagnosis was categorized according to the Agency for Healthcare Research and Quality Healthcare Utilization Project Clinical Classifications Software Single-Level Diagnosis Categories.

### Association Between Market AMC Presence and Mortality for Patients Hospitalized at Non-AMCs

**Figure 2.**
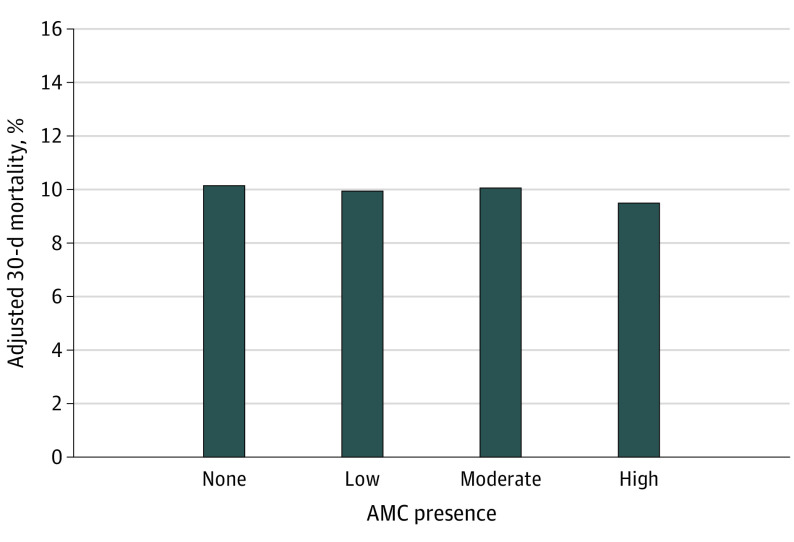
Variation in 30-Day Mortality by Market-Level Academic Medical Center (AMC) Presence, Adjusted for Patient and Market Characteristics Linear probability model incorporates year, hospital random effects, hospital region, principal discharge diagnosis, patient characteristics (age, sex, and Medicaid eligibility) and key market characteristics (total population, median income, percentage of Black residents, physicians per 100 000 population, hospital full-time equivalent per 1000 population, and median income). Graph was created using data from the Centers for Medicare & Medicaid Services (CMS) Master Beneficiary Summary File (2015-2017), 100% Medicare Inpatient File, the Dartmouth Atlas, the CMS Public Use Files, and the American Community Survey (2011-2015). Adjusted 30-day mortality was 10.1% for hospital referral regions (HRRs) with no AMC, 9.9% for HRRs with low AMC presence, 10.1% for HRRs with moderate AMC presence, and 9.5% for HRRs with high AMC presence.

Coefficients for all variables in the models adjusting for patient and hospital characteristics are presented in eTable 2 in Supplement 1. Effect size estimates are expressed in absolute percentage points generated from the linear probability model and are presented for each model in eTable 3 in Supplement 1. In the unadjusted model, compared with markets with no AMC presence, there was a significant association with lower mortality for treatment in a market with high (absolute difference, −1.1%; 95% CI, −1.4% to −0.8%; *P* < .001), moderate (absolute difference, −0.5%; 95% CI, −0.7% to −0.3%; *P* < .001), and low (absolute difference, −0.5%; 95% CI, −0.7% to −0.4%; *P* < .001) AMC presence. These differences persisted after adjusting for patient characteristics (eTable 3 in Supplement 1). After further adjusting for market characteristics, treatment in a market with high AMC presence was associated with 0.7% lower mortality compared with a market with no AMCs (9.5% vs 10.1%; absolute difference,−0.7%; 95% CI, −1.0 to −0.4%; *P* < .001). Treatment in a market with low AMC presence was associated with a −0.21% absolute difference in mortality at 30 days compared with a market with no AMCs (9.9% vs 10.1%; 95% CI, −0.4% to −0.1%; *P* = .006). There was no significant difference between markets with a moderate AMC presence vs those with no AMCs (absolute difference, −0.1%; 95% CI, −0.3% to 0.1%; *P* = .39). Adjusted means by group are shown in Figure 2 and eFigure 3 in Supplement 1.

Our results were similar for 90-day mortality. Compared with markets with no AMC presence, treatment at a non-AMC in markets with low, moderate, and high AMC presence with associated lower 90-day mortality in the unadjusted and patient characteristics models (eTable 3 in Supplement 1). After incorporating market characteristics, treatment in a market with high AMC presence was associated with an −0.8% absolute difference in 90-day mortality (16.1% vs 16.9%; 95% CI, −1.2% to −0.4%; *P* < .001) but there was no association for low or moderate AMC presence. Adjusted means by model are shown in eFigure 4 in Supplement 1.

### Association Between Market AMC Presence and Total HDAH

For all models, adjusted differences among market groups are presented in eTable 4 in Supplement 1, and adjusted mean outcomes by market group are presented in eFigures 5 and 6 in Supplement 1. There were few differences in the unadjusted models. Adjusting for patient characteristics, treatment in a market with high and low, but not moderate, AMC presence was associated a greater number of HDAH at 30 days (eTable 4 in Supplement 1). After further adjusting for market characteristics, these associations were smaller but remained significant. Markets with high AMC presence had 16.49 adjusted HDAH vs 16.12 for markets with no AMC presence (difference, 0.38 HDAH; 95% CI, 0.11 to 0.64 HDAH; *P* = .005) (eFigure 5 in Supplement 1). Markets with low AMC presence had 16.32 HDAH vs 16.12 HDAH for markets with no AMC presence (difference, 0.21 HDAH; 95% CI, 0.07 to 0.34 HDAH; *P* = .003). For 90-day HDAH, treatment in a market with high AMC presence was associated with 1.25 more HDAH compared with markets with no AMC presence (61.08 vs 59.83 HDAH; 95% CI, 0.58 to 1.92 HDAH; *P* < .001), and treatment in a market with low AMC presence was associated with 0.41 additional HDAH (95% CI, 0.07 to 0.76 HDAH; *P* = .02) in the final model incorporating market characteristics. However, there was no association for markets with moderate AMC presence (eFigure 6 in Supplement 1).

### Secondary Analyses

To address the concern that patients with less-severe conditions reside in markets with high AMC presence and, therefore, have lower mortality, we checked for analogous differences in mortality among patients seen at AMCs (eFigure 7 in Supplement 1). There was no significant association between market-level AMC presence and outcomes for patients treated at AMCs in any of the mortality models (eTable 4 in Supplement 1), suggesting that patients are equally healthy in markets with high and low AMC presence.

When examining AMC presence as a continuous variable, the locally estimated scatterplot smoothing curve revealed a small decline in 30-day mortality with increasing AMC presence (eFigure 8 in Supplement 1), consistent with our primary finding. After adjusting for patient and market characteristics, each 1% increase in market-level AMC presence was associated with −0.20% difference in 30-day mortality for patients hospitalized at non-AMCs (95% CI, −0.23% to −0.07%; *P* < .001).

As noted in Table 1, the percentage of hospitalizations at non-AMCs ending in transfer was 2.1% in markets with no and low AMC presence, 2.9% in markets with moderate AMC presence, and 3.1% in markets with high AMC presence. After incorporating HRR-level transfer rates as a covariate, the association between hospitalization in a market with high AMC presence and lower 30-day mortality persisted but was lesser in magnitude (9.7% vs 10.1%; absolute difference, −0.4%; 95% CI, −0.7% to −0.1%; *P* = .005) (eTable 3 and eFigure 3 in Supplement 1) compared with the model only accounting for patient and market characteristics. The impact on accounting for transfers was similar for the outcome of 90-day mortality (16.3% vs 16.8%; difference, −0.5%; 95% CI, −0.9% to −0.1%; *P* = .01) (eTable 3 and eFigure 4 in Supplement 1).

## Discussion

In a national cohort study of more than 22 million hospitalizations among traditional Medicare beneficiaries from 2015 to 2017, patients hospitalized at non-AMCs had lower 30-day and 90-day mortality and more HDAH at 30 and 90 days when they received care in markets with greater AMC presence. Adjusting for patient characteristics increased this association relative to the unadjusted model, which persisted even when accounting for other demographic and health system factors that differed among the market groups. In contrast, we found no association between market-level AMC presence and outcomes for patients treated at AMCs themselves. Taken together, these results suggest a spillover effect of AMCs on outcomes for neighboring community hospitals and that the benefit of AMCs for the broader community may be greater than is traditionally recognized.

There are multiple potential mechanisms by which AMCs may improve outcomes for patients in community hospitals. Patients hospitalized at non-AMCs in regions with higher AMC presence were more often transferred to another short-term general hospital for ongoing care. It is possible that greater AMC availability in these markets allowed non-AMCs to transfer patients who would most benefit from advanced capabilities at tertiary centers.^33^ Prior research has suggested that transfer is associated with better outcomes for selected conditions^32^ and that the failure to transfer in a timely fashion represents a source of inequity.^34,35^ When we accounted for market-level differences in transfer rates, the association between high AMC presence and lower mortality for patients hospitalized at non-AMCs was smaller but remained significant. These findings suggest that variation in tertiary care transfer likely accounts for some, but not all, of the possible mortality benefit of receiving care in a market with greater AMC presence for patients hospitalized at non-AMCs.

Markets with the greatest AMC presence had more nurses and physicians per capita. There is evidence that physicians tend to practice in close geographic proximity to where they trained,^36^ with more than one-half of residency graduates historically practicing in the same state after graduation.^37,38^ Thus, it is possible that the presence of an AMC may lead to a more robust physician supply and better patient outcomes at community hospitals. Alternatively, it may be that a number of formal and informal affiliations between AMCs and non-AMCs within the same market leads to diffusion of knowledge, innovation, and even sharing of clinicians who work at multiple sites.^39,40^ For key serious conditions, such as a stroke, AMCs have been shown to promote evidence-based practice and improve patient outcomes at community hospitals.^41^

This study extends prior work examining the association of AMCs with acute care outcomes. Multiple studies^13,14,15,16,17,18,19,20,21^ have suggested that patients treated at AMCs have better outcomes across the spectrum of patient severity. To our knowledge, this is the first study to systematically examine the potential indirect clinical benefits of receiving care at a nonteaching hospital with greater proximity to 1 or more AMCs across a wide range of conditions.

This study is consistent with other studies demonstrating geographic disparities in health care access,^42^ particularly for key specialty services.^12,43^ These data highlight the degree to which AMCs tend to be concentrated, with rural regions having less access to AMC services on average. Thus, identifying strategies by which AMCs may enhance care for patients in rural and other remote locations^44,45,46^ has the potential to improve health outcomes for underserved populations and widen the reach of the nation’s academic health care institutions.

### Limitations

The observational nature of this study precludes assigning a causal relationship between market-level AMC presence and outcomes. Markets with differing degrees of AMC presence may differ on other key characteristics of interest. We addressed these concerns by specifying models incorporating observable indicators of patient severity and characteristics of the markets themselves. However, it is possible that there are residual patient and market confounders that may impact our results. To address this, we repeated our models among patients hospitalized at AMCs themselves, hypothesizing that if market factors were the key factors associated with any differential patient outcomes—rather than spillover effects of AMCs within the market—then we would see analogous trends for patients hospitalized at AMCs. In addition, although this study examines all hospitalizations among traditional Medicare beneficiaries, it is possible that the results may differ for other populations. Furthermore, we chose a linear probability model because the results can be interpreted as absolute differences in percentages between category of HRR-level AMC presence. Absolute differences are more interpretable than relative differences reflected in odds ratios from a logistic regression model. However, the linear probability model has technical disadvantages because the model can estimate probabilities below 0 or above 1 and does not allow for unequal variances.

## Conclusions

Among Medicare beneficiaries hospitalized at non-AMCs from 2015 to 2017, this cohort study found that treatment in a market with greater AMC presence was associated with lower mortality and a greater number of HDAH at 30 and 90 days. This association persisted after accounting for differences in patient and market characteristics, including variation in rates of interhospital transfer. These findings suggest that the impact of AMCs on clinical outcomes extends beyond direct patient care and that there are spillover effects of AMCs on outcomes for patients in the broader health care market.

## Notes

Supplement 1. **eTable 1.** SAS Code for Main Models Adjusting for Year, Hospital Random Effects, Hospital Region, Diagnosis Related Group (DRG) Weight, Patient Characteristics and Market Characteristics **eFigure 1.** Histogram Demonstrating the Distribution of Academic Medical Centers (AMCs) Among Healthcare Markets (Hospital Referral Regions) in the United States in 2015 **eFigure 2.** Variation Among Healthcare Markets With Respect to the Percentage of Hospitalizations in 2015-2017 Managed at Academic Medical Centers **eTable 2.** Association Between Market AMC Presence and 30-Day Mortality for Patients Hospitalized at Non-AMCs **eTable 3.** Comparison of 30- and 90-Day Mortality at Non-Academic Medical Centers (Non-AMCs) by Category of Market-Level AMC Presence and Adjustment Model **eFigure 3.** Variation in 30-Day Mortality Among Patients Hospitalized at Non-AMCs, Stratified by Market-Level AMC Presence, by Adjustment Model **eFigure 4.** Variation in 90-Day Mortality Among Patients Hospitalized at Non-AMCs, Stratified by Market-Level AMC Presence, by Adjustment Model **eTable 4.** Comparison of 30- and 90-Day Healthy Days at Home (HDAH) at Non-Academic Medical Centers (Non-AMCs) by Category of Market-Level AMC Presence and Adjustment Model **eFigure 5.** Variation in 30-Day Healthy Days at Home (HDAH) Among Patients Hospitalized at Non-AMCs, Stratified by Market-Level AMC Presence and Adjustment Model **eFigure 6.** Variation in 90-Day Healthy Days at Home (HDAH) Among Patients Hospitalized at Non-AMCs, Stratified by Market-Level AMC Presence and Adjustment Model **eFigure 7.** Association Between Market-Level AMC Presence and Mortality for Patients Treated AMCs, Stratified by Adjustment Model **eTable 5.** Comparison of 30- and 90-Day Mortality at Academic Medical Centers (AMCs) by Category of Market-Level AMC Presence and Adjustment Model **eFigure 8.** A LOESS (Locally Estimated Scatterplot Smoothing) Plot of the Relationship Between Unadjusted 30-Day Mortality for Patients Treated at Non-AMCs and HRR-Level AMC Presence

Supplement 2.
Data Sharing Statement
