# Supplementary material for: Association of Academic Medical Center Presence With Clinical Outcomes at Neighboring Community Hospitals Among Medicare Beneficiaries
Source: JAMA Netw Open. 2023 Feb 1;6(2):e2254559. doi: 10.1001/jamanetworkopen.2022.54559 (PMC9892959; doi:10.1001/jamanetworkopen.2022.54559)
Supplement: Supplement 1. — eTable 1. SAS Code for Main Models Adjusting for Year, Hospital Random Effects, Hospital Region, Diagnosis Related Group (DRG) Weight, Patient Characteristics and Market Characteristics eFigure 1. Histogram Demonstrating the Distribution of Academic Medical Centers (AMCs) Among Healthcare Markets (Hospital Referral Regions) in the United States in 2015 eFigure 2. Variation Among Healthcare Markets With Respect to the Percentage of Hospitalizations in 2015-2017 Managed at Academic Medical Centers eTable 2. Association Between Market AMC Presence and 30-Day Mortality for Patients Hospitalized at Non-AMCs eTable 3. Comparison of 30- and 90-Day Mortality at Non-Academic Medical Centers (Non-AMCs) by Category of Market-Level AMC Presence and Adjustment Model eFigure 3. Variation in 30-Day Mortality Among Patients Hospitalized at Non-AMCs, Stratified by Market-Level AMC Presence, by Adjustment Model eFigure 4. Variation in 90-Day Mortality Among Patients Hospitalized at Non-AMCs, Stratified by Market-Level AMC Presence, by Adjustment Model eTable 4. Comparison of 30- and 90-Day Healthy Days at Home (HDAH) at Non-Academic Medical Centers (Non-AMCs) by Category of Market-Level AMC Presence and Adjustment Model eFigure 5. Variation in 30-Day Healthy Days at Home (HDAH) Among Patients Hospitalized at Non-AMCs, Stratified by Market-Level AMC Presence and Adjustment Model eFigure 6. Variation in 90-Day Healthy Days at Home (HDAH) Among Patients Hospitalized at Non-AMCs, Stratified by Market-Level AMC Presence and Adjustment Model eFigure 7. Association Between Market-Level AMC Presence and Mortality for Patients Treated AMCs, Stratified by Adjustment Model eTable 5. Comparison of 30- and 90-Day Mortality at Academic Medical Centers (AMCs) by Category of Market-Level AMC Presence and Adjustment Model eFigure 8. A LOESS (Locally Estimated Scatterplot Smoothing) Plot of the Relationship Between Unadjusted 30-Day Mortality for Patients Treated at Non-AMCs and HRR-Level AMC Presence [file jamanetwopen-e2254559-s001.pdf]

Supplemental Online Content

Burke LG, Burke RC, Orav EJ, Duggan CE, Figueroa JF, Jha AK. Association of academic medical center presence with clinical outcomes at neighboring community hospitals among Medicare beneficiaries. *JAMA Netw Open*. 2023;6(2):e2254559. doi:10.1001/jamanetworkopen.2022.54559

eTable 1. SAS Code for Main Models Adjusting for Year, Hospital Random Effects, Hospital Region, Diagnosis Related Group (DRG) Weight, Patient Characteristics and Market Characteristics
eFigure 1. Histogram Demonstrating the Distribution of Academic Medical Centers (AMCs) Among Healthcare Markets (Hospital Referral Regions) in the United States in 2015
eFigure 2. Variation Among Healthcare Markets With Respect to the Percentage of Hospitalizations in 2015-2017 Managed at Academic Medical Centers
eTable 2. Association Between Market AMC Presence and 30-Day Mortality for Patients Hospitalized at Non-AMCs
eTable 3. Comparison of 30- and 90-Day Mortality at Non-Academic Medical Centers (Non-AMCs) by Category of Market-Level AMC Presence and Adjustment Model
eFigure 3. Variation in 30-Day Mortality Among Patients Hospitalized at Non-AMCs, Stratified by Market-Level AMC Presence, by Adjustment Model
eFigure 4. Variation in 90-Day Mortality Among Patients Hospitalized at Non-AMCs, Stratified by Market-Level AMC Presence, by Adjustment Model
eTable 4. Comparison of 30- and 90-Day Healthy Days at Home (HDAH) at Non-Academic Medical Centers (Non-AMCs) by Category of Market-Level AMC Presence and Adjustment Model
eFigure 5. Variation in 30-Day Healthy Days at Home (HDAH) Among Patients Hospitalized at Non-AMCs, Stratified by Market-Level AMC Presence and Adjustment Model
eFigure 6. Variation in 90-Day Healthy Days at Home (HDAH) Among Patients Hospitalized at Non-AMCs, Stratified by Market-Level AMC Presence and Adjustment Model
eFigure 7. Association Between Market-Level AMC Presence and Mortality for Patients Treated AMCs, Stratified by Adjustment Model
eTable 5. Comparison of 30- and 90-Day Mortality at Academic Medical Centers (AMCs) by Category of Market-Level AMC Presence and Adjustment Model
eFigure 8. A LOESS (Locally Estimated Scatterplot Smoothing) Plot of the Relationship Between Unadjusted 30-Day Mortality for Patients Treated at Non-AMCs and HRR-Level AMC Presence

This supplemental material has been provided by the authors to give readers additional information about their work.

© 2023 Burke LG et al. *JAMA Network Open.*

eTable 1. SAS Code for Main Models Adjusting for Year, Hospital Random Effects, Hospital Region, Diagnosis Related Group (DRG) Weight, Patient Characteristics and Market Characteristics

OUTCOME	NOTES	CODE
**30-day Mortality**	Otcc_autism was removed due to very small prevalence which was causing the model to not converge.	proc mixed data=death30 plots=none noclprint;<br>class amcpresence (ref='0') hosp_reg4 ccs_category_disch provider sex dual ccw_alzhdmta ccw_ami ccw_anemia ccw_asthma ccw_atrialfb ccw_cataract ccw_chf ccw_chrnkidn ccw_cncrbrst ccw_cncrclrc ccw_cncrendm ccw_cncrlung ccw_cncrprst ccw_copd ccw_diabetes ccw_glaucoma ccw_hipfrac ccw_hyperl ccw_hyperp ccw_hypert ccw_hypoth ccw_ischmcht ccw_osteoprs ccw_ra_oa ccw_STRKETIA depression otcc_ACP otcc_ALCO otcc_ANXI otcc_BIPL otcc_BRAINJ otcc_CERPAL otcc_CYSFIB otcc_EPILEP otcc_FIBRO otcc_HEARIM otcc_HEPVIRAL otcc_HIVAIDS otcc_INTDIS otcc_LEADIS otcc_LEUKLYMPH otcc_LIVER otcc_MIGRAINE otcc_MOBIMP otcc_MULSCL otcc_MUSDYS otcc_OBESITY otcc_OTHDEL otcc_PSDS otcc_PTRA otcc_PVD otcc_SCD otcc_SCHI otcc_SCHIOT otcc_SPIBIF otcc_SPIINJ otcc_TOBA otcc_ULCERS otcc_VISUAL;<br>model mort30=amcpresence hosp_reg4 ccs_category_disch DRGWTAMT age sex dual year ccw_alzhdmta ccw_ami ccw_anemia ccw_asthma ccw_atrialfb ccw_cataract ccw_chf ccw_chrnkidn ccw_cncrbrst ccw_cncrclrc ccw_cncrendm ccw_cncrlung ccw_cncrprst ccw_copd ccw_diabetes ccw_glaucoma ccw_hipfrac ccw_hyperl ccw_hyperp ccw_hypert ccw_hypoth ccw_ischmcht ccw_osteoprs ccw_ra_oa ccw_STRKETIA depression otcc_ACP otcc_ALCO otcc_ANXI otcc_BIPL otcc_BRAINJ otcc_CERPAL otcc_CYSFIB otcc_EPILEP otcc_FIBRO otcc_HEARIM otcc_HEPVIRAL otcc_HIVAIDS otcc_INTDIS otcc_LEADIS otcc_LEUKLYMPH otcc_LIVER otcc_MIGRAINE otcc_MOBIMP otcc_MULSCL otcc_MUSDYS otcc_OBESITY otcc_OTHDEL otcc_PSDS otcc_PTRA otcc_PVD otcc_SCD otcc_SCHI otcc_SCHIOT otcc_SPIBIF otcc_SPIINJ otcc_TOBA otcc_ULCERS otcc_VISUAL HRRpctblack hrrpopulation hrrdocs_per1k hrrhospfte_per1k hrrmedianincome/ solution cl;<br>random int / subject=provider;<br>lsmeans amcpresence / om cl;<br>run;
**90-day Mortality**		proc mixed data=death90 plots=none noclprint;<br>class amcpresence (ref='0') hosp_reg4 ccs_category_disch provider sex dual ccw_alzhdmta ccw_ami ccw_anemia ccw_asthma ccw_atrialfb ccw_cataract ccw_chf ccw_chrnkidn ccw_cncrbrst ccw_cncrclrc ccw_cncrendm ccw_cncrlung ccw_cncrprst ccw_copd ccw_diabetes ccw_glaucoma ccw_hipfrac ccw_hyperl ccw_hyperp ccw_hypert ccw_hypoth ccw_ischmcht ccw_osteoprs ccw_ra_oa ccw_STRKETIA depression otcc_ACP otcc_ALCO otcc_ANXI otcc_AUTISM otcc_BIPL otcc_BRAINJ otcc_CERPAL otcc_CYSFIB otcc_EPILEP otcc_FIBRO otcc_HEARIM otcc_HEPVIRAL otcc_HIVAIDS otcc_INTDIS otcc_LEADIS

© 2023 Burke LG et al. *JAMA Network Open.*

		otcc_LEUKLYMPH otcc_LIVER otcc_MIGRAINE otcc_MOBIMP otcc_MULSCL otcc_MUSDYS otcc_OBESITY otcc_OTHDEL otcc_PSDS otcc_PTRA otcc_PVD otcc_SCD otcc_SCHI otcc_SCHIOT otcc_SPIBIF otcc_SPIINJ otcc_TOBA otcc_ULCERS otcc_VISUAL;<br>model mort90=amcpresence hosp_reg4 ccs_category_disch DRGWTAMT age sex dual year ccw_alzhdmta ccw_ami ccw_anemia ccw_asthma ccw_atrialfb ccw_cataract ccw_chf ccw_chrnkidn ccw_cncrbrst ccw_cncrclrc ccw_cncrendm ccw_cncrlung ccw_cncrprst ccw_copd ccw_diabetes ccw_glaucoma ccw_hipfrac ccw_hyperl ccw_hyperp ccw_hypert ccw_hypoth ccw_ischmcht ccw_osteoprs ccw_ra_oa ccw_STRKETIA depression otcc_ACP otcc_ALCO otcc_ANXI otcc_AUTISM otcc_BIPL otcc_BRAINJ otcc_CERPAL otcc_CYSFIB otcc_EPILEP otcc_FIBRO otcc_HEARIM otcc_HEPVIRAL otcc_HIVAIDS otcc_INTDIS otcc_LEADIS otcc_LEUKLYMPH otcc_LIVER otcc_MIGRAINE otcc_MOBIMP otcc_MULSCL otcc_MUSDYS otcc_OBESITY otcc_OTHDEL otcc_PSDS otcc_PTRA otcc_PVD otcc_SCD otcc_SCHI otcc_SCHIOT otcc_SPIBIF otcc_SPIINJ otcc_TOBA otcc_ULCERS otcc_VISUAL HRRpctblack hrrpopulation hrrdocs_per1k hrrhospfte_per1k hrrmedianincome/ solution cl;<br>random int / subject=provider;<br>lsmeans amcpresence / om cl;<br>run;
**30-day HDAH**	Otcc_autism was removed due to very small prevalence which was causing the model to not converge.	proc mixed data=hdah30 plots=none noclprint;<br>class amcpresence (ref='0') hosp_reg4 ccs_category_disch provider sex dual ccw_alzhdmta ccw_ami ccw_anemia ccw_asthma ccw_atrialfb ccw_cataract ccw_chf ccw_chrnkidn ccw_cncrbrst ccw_cncrclrc ccw_cncrendm ccw_cncrlung ccw_cncrprst ccw_copd ccw_diabetes ccw_glaucoma ccw_hipfrac ccw_hyperl ccw_hyperp ccw_hypert ccw_hypoth ccw_ischmcht ccw_osteoprs ccw_ra_oa ccw_STRKETIA depression otcc_ACP otcc_ALCO otcc_ANXI otcc_AUTISM otcc_BIPL otcc_BRAINJ otcc_CERPAL otcc_CYSFIB otcc_EPILEP otcc_FIBRO otcc_HEARIM otcc_HEPVIRAL otcc_HIVAIDS otcc_INTDIS otcc_LEADIS otcc_LEUKLYMPH otcc_LIVER otcc_MIGRAINE otcc_MOBIMP otcc_MULSCL otcc_MUSDYS otcc_OBESITY otcc_OTHDEL otcc_PSDS otcc_PTRA otcc_PVD otcc_SCD otcc_SCHI otcc_SCHIOT otcc_SPIBIF otcc_SPIINJ otcc_TOBA otcc_ULCERS otcc_VISUAL;<br>model hdah=amcpresence hosp_reg4 ccs_category_disch DRGWTAMT age sex dual year ccw_alzhdmta ccw_ami ccw_anemia ccw_asthma ccw_atrialfb ccw_cataract ccw_chf ccw_chrnkidn ccw_cncrbrst ccw_cncrclrc ccw_cncrendm ccw_cncrlung ccw_cncrprst ccw_copd ccw_diabetes ccw_glaucoma ccw_hipfrac ccw_hyperl ccw_hyperp ccw_hypert ccw_hypoth ccw_ischmcht ccw_osteoprs ccw_ra_oa ccw_STRKETIA depression otcc_ACP otcc_ALCO otcc_ANXI otcc_AUTISM otcc_BIPL otcc_BRAINJ otcc_CERPAL otcc_CYSFIB otcc_EPILEP<br>otcc_FIBRO otcc_HEARIM otcc_HEPVIRAL otcc_HIVAIDS otcc_INTDIS otcc_LEADIS otcc_LEUKLYMPH otcc_LIVER

© 2023 Burke LG et al. *JAMA Network Open.*

		otcc_MIGRAINE otcc_MOBIMP otcc_MULSCL otcc_MUSDYS otcc_OBESITY otcc_OTHDEL otcc_PSDS otcc_PTRA otcc_PVD otcc_SCD otcc_SCHI otcc_SCHIOT otcc_SPIBIF otcc_SPIINJ otcc_TOBA otcc_ULCERS otcc_VISUAL HRRpctblack hrrpopulation hrrdocs_per1k hrrhospfte_per1k hrrmedianincome/ solution cl;<br>random int / subject=provider;<br>lsmeans amcpresence / om cl;<br>run;
**90-day HDAH**		proc mixed data=hdah90 plots=none noclprint;<br>class amcpresence (ref='0') hosp_reg4 ccs_category_disch provider sex dual ccw_alzhdmta ccw_ami ccw_anemia ccw_asthma ccw_atrialfb ccw_cataract ccw_chf ccw_chrnkidn ccw_cncrbrst ccw_cncrclrc ccw_cncrendm ccw_cncrlung ccw_cncrprst ccw_copd ccw_diabetes ccw_glaucoma ccw_hipfrac ccw_hyperl ccw_hyperp ccw_hypert ccw_hypoth ccw_ischmcht ccw_osteoprs ccw_ra_oa ccw_STRKETIA depression otcc_ACP otcc_ALCO otcc_ANXI otcc_BIPL otcc_BRAINJ otcc_CERPAL otcc_CYSFIB otcc_EPILEP otcc_FIBRO otcc_HEARIM otcc_HEPVIRAL otcc_HIVAIDS otcc_INTDIS otcc_LEADIS otcc_LEUKLYMPH otcc_LIVER otcc_MIGRAINE otcc_MOBIMP otcc_MULSCL otcc_MUSDYS otcc_OBESITY otcc_OTHDEL otcc_PSDS otcc_PTRA otcc_PVD otcc_SCD otcc_SCHI otcc_SCHIOT otcc_SPIBIF otcc_SPIINJ otcc_TOBA otcc_ULCERS otcc_VISUAL;<br>model hdah=amcpresence hosp_reg4 ccs_category_disch DRGWTAMT age sex dual year ccw_alzhdmta ccw_ami ccw_anemia ccw_asthma ccw_atrialfb ccw_cataract ccw_chf ccw_chrnkidn ccw_cncrbrst ccw_cncrclrc ccw_cncrendm ccw_cncrlung ccw_cncrprst ccw_copd ccw_diabetes ccw_glaucoma ccw_hipfrac ccw_hyperl ccw_hyperp ccw_hypert ccw_hypoth ccw_ischmcht ccw_osteoprs ccw_ra_oa ccw_STRKETIA depression otcc_ACP otcc_ALCO otcc_ANXI otcc_BIPL otcc_BRAINJ otcc_CERPAL otcc_CYSFIB otcc_EPILEP otcc_FIBRO otcc_HEARIM otcc_HEPVIRAL otcc_HIVAIDS otcc_INTDIS otcc_LEADIS otcc_LEUKLYMPH otcc_LIVER otcc_MIGRAINE otcc_MOBIMP otcc_MULSCL otcc_MUSDYS otcc_OBESITY otcc_OTHDEL otcc_PSDS otcc_PTRA otcc_PVD otcc_SCD otcc_SCHI otcc_SCHIOT otcc_SPIBIF otcc_SPIINJ otcc_TOBA otcc_ULCERS otcc_VISUAL HRRpctblack hrrpopulation hrrdocs_per1k hrrhospfte_per1k hrrmedianincome/ solution cl;<br>random int / subject=provider;<br>lsmeans amcpresence / om cl;<br>run;

© 2023 Burke LG et al. *JAMA Network Open.*

eFigure 1. Histogram Demonstrating the Distribution of Academic Medical Centers (AMCs) Among Healthcare Markets (Hospital Referral Regions) in the United States in 2015

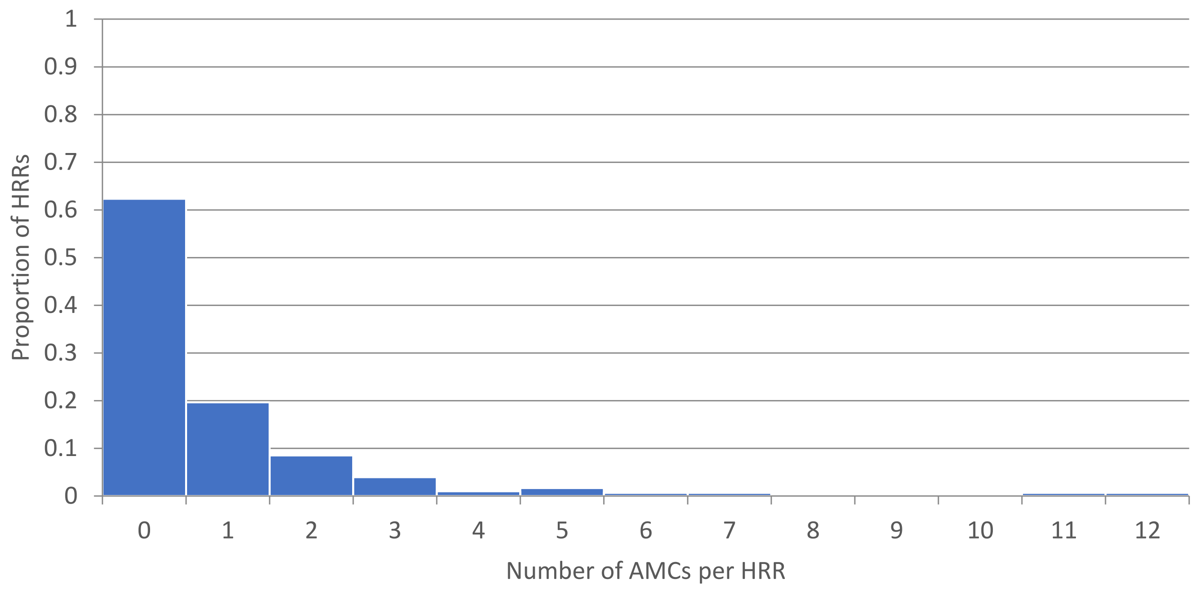

© 2023 Burke LG et al. *JAMA Network Open.*

eFigure 2. Variation Among Healthcare Markets* With Respect to the Percentage of Hospitalizations in 2015-2017 Managed at Academic Medical Centers

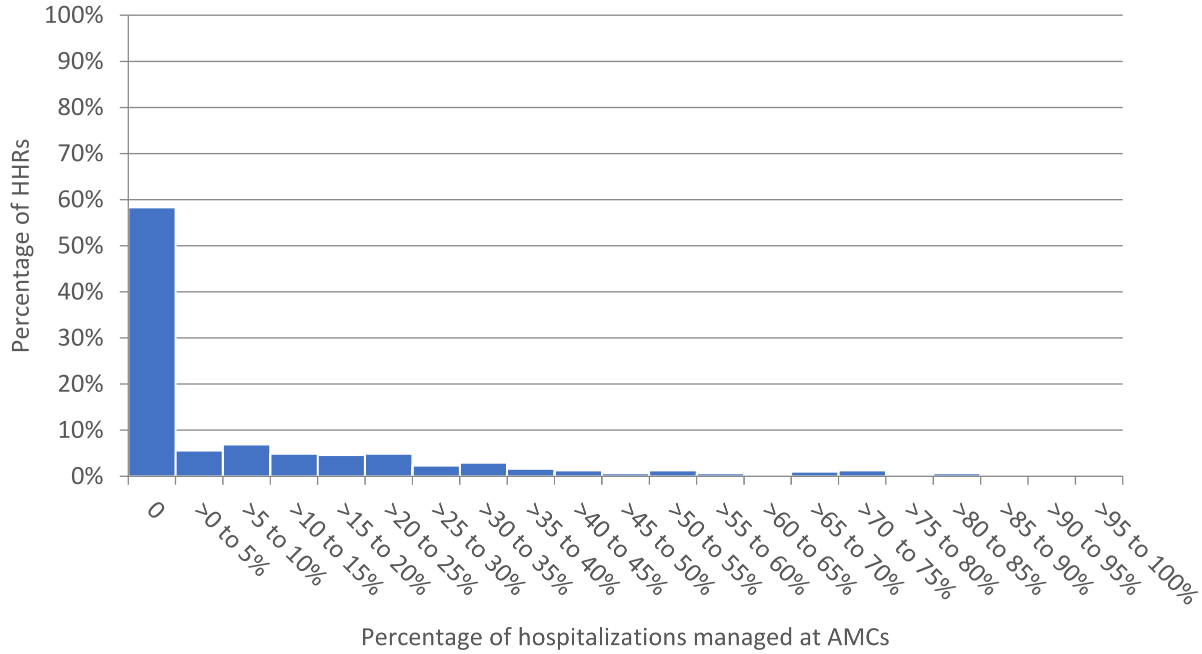

*Healthcare markets defined by the Dartmouth Atlas Hospital Referral Regions (HRRs). Academic Medical Centers (AMCs) were defined by the Intern/Resident to Bed Ratio (IRB) obtained from the Medicare Cost reports and was identified for each hospital yearly. Hospitals with an IRB of 0.25 or greater were considered AMCs.

© 2023 Burke LG et al. *JAMA Network Open.*

eTable 2. Association Between Market AMC Presence and 30-Day Mortality for Patients Hospitalized at Non-AMCs

30-DAY MORTALITY					
**Covariate**	**Estimate**	**Standard Error**	**P-Value**	**Lower CI**	**Upper CI**
Intercept	8.32	0.1704	<.0001	7.986	8.654
**HRR Category of AMC Presence**					
Low AMC Presence	-0.00211	0.000769	0.006	-0.00362	-0.0006
Moderate AMC Presence	-0.00092	0.001067	0.39	-0.00301	0.001168
High AMC Presence	-0.00656	0.001502	<.0001	-0.00951	-0.00362
No AMC Presence	REF	REF	REF	REF	REF
**Region**					
Northeast	0.00395	0.001282	0.002	0.001438	0.006462
Midwest	0.00644	0.001017	<.0001	0.004447	0.008433
South	0.003829	0.001015	0.0002	0.001839	0.005819
West	REF	REF	REF	REF	REF
**Patient/Hospitalization Characteristics**					
Principal Discharge Diagnosis Category*	*	*	*	*	*
DRG weight amount	0.01453	0.000056	<.0001	0.01442	0.01464
Age (years)	0.00326	9.77E-06	<.0001	0.003241	0.003279
Male	0.01368	0.000179	<.0001	0.01333	0.01403
Female	0	.	.	.	.
Not Medicaid Eligible	0.001728	0.000175	<.0001	0.001386	0.002071
Medicaid Eligible	0	.	.	.	.
Year	-0.0043	0.000084	<.0001	-0.00446	-0.00413
Beneficiary Chronic Conditions (CCW and other related)*	*	*	*	*	*
**HRR Characteristics**					
HRR Percentage Black Population	-0.00764	0.003538	0.031	-0.01457	-0.0007
HRR Total Population	-1.20E-09	0	<.0001	.	.
HRR Doctors Per 100k Population	-0.00001	0.000015	0.35	-0.00004	0.000015
HRR Hospital FTE Per 1k Population	0.000488	0.000127	0.0001	0.000239	0.000737
HRR Median Income	-1.18E-07	0	<.0001	.	.

© 2023 Burke LG et al. *JAMA Network Open.*

90-DAY MORTALITY					
**Covariate**	**Estimate**	**Standard Error**	**P-Value**	**Lower CI**	**Upper CI**
Intercept	15.4787	0.2179	<.0001	15.0514	15.9059
**HRR Category of AMC Presence**					
Low AMC Presence	-0.00155	0.000998	0.12	-0.00351	0.000407
Moderate AMC Presence	-0.00059	0.001385	0.67	-0.0033	0.002129
High AMC Presence	-0.00785	0.001951	<.0001	-0.01168	-0.00403
No AMC Presence	REF	REF	REF	REF	REF
**Region**					
Northeast	0.004823	0.001665	0.004	0.001561	0.008086
Midwest	0.007051	0.001318	<.0001	0.004467	0.009635
South	0.003872	0.001318	0.003	0.001289	0.006455
West	REF	REF	REF	REF	REF
**Patient/Hospitalization Characteristics**					
Principal Discharge Diagnosis Category*	*	*	*	*	*
DRG weight amount	0.0194	0.000071	<.0001	0.01926	0.01954
Age (years)	0.004557	0.000012	<.0001	0.004533	0.004581
Male	0.02023	0.000225	<.0001	0.01979	0.02067
Female	REF	REF	REF	REF	REF
Not Medicaid Eligible	0.004244	0.000219	<.0001	0.003814	0.004673
Medicaid Eligible	REF	REF	REF	REF	REF
Year	-0.00782	0.000108	<.0001	-0.00803	-0.00761
Beneficiary Chronic Conditions (CCW and other related)*	*	*	*	*	*
**HRR Characteristics**					
HRR Percentage Black Population	-0.00055	0.004589	0.90	-0.00955	0.008444
HRR Total Population	-1.60E-09	0	<.0001	.	.
HRR Doctors Per 100k Population	-0.00003	0.000019	0.14	-0.00007	8.95E-06
HRR Hospital FTE Per 1k Population	0.000729	0.000164	<.0001	0.000407	0.001052
HRR Median Income	-1.41E-07	0	<.0001	.	.

© 2023 Burke LG et al. *JAMA Network Open.*

30-DAY HDAH					
Covariate	Estimate	Standard Error	P-Value	Lower CI	Upper CI
Intercept	-717	12.38	<.0001	-740.96	-692.42
**HRR Category of AMC Presence**					
Low AMC Presence	0.208	0.069	0.0025	0.073	0.343
Moderate AMC Presence	0.003	0.095	0.98	-0.184	0.190
High AMC Presence	0.375	0.135	0.005	0.111	0.640
No AMC Presence	REF	REF	REF	REF	REF
**Region**					
Northeast	-0.567	0.115	<.0001	-0.792	-0.342
Midwest	-0.719	0.090	<.0001	-0.896	-0.542
South	0.016	0.091	0.8627	-0.162	0.194
West	REF	REF	REF	REF	REF
**Patient/Hospitalization Characteristics**					
Principal Discharge Diagnosis Category*	*	*	*	*	*
DRG weight amount	-1.60	0.0041	<.0001	-1.60	-1.59
Age (years)	-0.17	0.0007	<.0001	-0.17	-0.17
Male	0.26	0.0131	<.0001	0.24	0.29
Female	REF	REF	REF	REF	REF
Not Medicaid Eligible	1.2364	0.01274	<.0001	1.2115	1.2614
Medicaid Eligible	REF	REF	REF	REF	REF
Year	0.3602	0.006131	<.0001	0.3482	0.3722
Beneficiary Chronic Conditions (CCW and other related)*	-1.60	0.0041	<.0001	-1.60	-1.59
**HRR Characteristics**					
HRR Percentage Black Population	0.1529	0.3151	0.6276	-0.4648	0.7705
HRR Total Population	6.03E-08	0	<.0001	.	.
HRR Doctors Per 100k Population	0.003606	0.001317	0.0062	0.001025	0.006187
HRR Hospital FTE Per 1k Population	-0.1016	0.01124	<.0001	-0.1236	-0.07957
HRR Median Income	-0.00001	3.43E-06	0.0002	-0.00002	-6.04E-06

© 2023 Burke LG et al. *JAMA Network Open.*

90-DAY HDAH					
**Covariate**	**Estimate**	**Standard Error**	**P-Value**	**Lower CI**	**Upper CI**
Intercept	-2313.8	37.4397	<.0001	-2387.2	-2240.39
**HRR Category of AMC Presence**					
Low AMC Presence	0.4128	0.1749	0.0182	0.07006	0.7555
Moderate AMC Presence	0.1284	0.2428	0.5968	-0.3474	0.6042
High AMC Presence	1.254	0.3421	0.0002	0.5836	1.9244
No AMC Presence	0	.	.	.	.
**Region**					
Northeast	-1.1253	0.2917	0.0001	-1.697	-0.5536
Midwest	-2.2266	0.2308	<.0001	-2.679	-1.7742
South	-0.2874	0.2309	0.2132	-0.7399	0.1651
West	0	.	.	.	.
**Patient/Hospitalization Characteristics**					
Principal Discharge Diagnosis Category*	*	*	*	*	*
DRG weight amount	-3.6234	0.01228	<.0001	-3.6474	-3.5993
Age (years)	-0.4584	0.002112	<.0001	-0.4625	-0.4542
Male	-0.5789	0.03898	<.0001	-0.6553	-0.5025
Female	0	.	.	.	.
Not Medicaid Eligible	4.4637	0.03774	<.0001	4.3897	4.5376
Medicaid Eligible	0	.	.	.	.
Year	1.1677	0.01855	<.0001	1.1314	1.2041
Beneficiary Chronic Conditions (CCW and other related)*	*	*	*	*	*
**HRR Characteristics**					
HRR Percentage Black Population	0.7391	0.8034	0.3576	-0.8355	2.3138
HRR Total Population	9.62E-08	0	<.0001	.	.
HRR Doctors Per 100k Population	0.01127	0.00335	0.0008	0.004699	0.01783
HRR Hospital FTE Per 1k Population	-0.2723	0.0288	<.0001	-0.3287	-0.2158
HRR Median Income	-0.00002	8.69E-06	0.076	-0.00003	1.61E-06

* Output for diagnosis categories and chronic conditions omitted due to length.

© 2023 Burke LG et al. *JAMA Network Open.*

eTable 3. Comparison of 30- and 90-Day Mortality at Non-Academic Medical Centers (Non-AMCs) by Category of Market-Level AMC Presence and Adjustment Model*

	**30-Day Mortality**		**90-Day Mortality**	
**UNADJUSTED**				
**AMC Presence**	**Absolute mortality difference (95% CI)**	**P-Value**	**Absolute mortality difference (95% CI)**	**P-Value**
No	REFERENCE		REFERENCE	
Low	-0.5% (-0.7% to 0.4%)	<.001	-0.4% (-0.6% to -0.3%)	<.001
Moderate	-0.5% (-0.7% to -0.3%)	<.001	-0.5% (-0.7% to -0.2%)	<.001
High AMC	-1.1% (-1.4 to -0.8%)	<.001	-1.0% (-1.3% to -0.6%)	<.001
**ADJUSTED FOR PATIENT CHARACTERISTICS**				
**AMC Presence**	**Absolute mortality difference (95% CI)**	**P-Value**	**Absolute mortality difference (95% CI)**	**P-Value**
No	REFERENCE		REFERENCE	
Low	-0.6% (-0.7% to -0.5%)	<.001	-0.7% (-0.8% to -0.5%)	<.001
Moderate	-0.4% (-0.6% to -0.2%)	<.001	-0.4% (-0.7% to -0.2%)	<.001
High	-1.1% (-1.3 to -0.8%)	<.001	-1.3% (-1.6% to -0.9%)	<.001
**ADJUSTED FOR MARKET CHARACTERISTICS**				
**AMC Presence**	**Absolute mortality difference (95% CI)**	**P-Value**	**Absolute mortality difference 95% CI**	**P-Value**
No	REFERENCE		REFERENCE	
Low	-0.2% (-0.4% to -0.1%)	0.006	-0.2% (-0.4% to 0.04%)	0.12
Moderate	-0.1% (-0.3% to 0.1%)	0.39	-0.1% (-0.3% to 0.2%)	0.67
High	-0.7% (-1.0% to -0.4%)	<.001	-0.8% (-1.2% to -0.4%)	<.001
**ADJUSTED FOR MARKET TRANFSER RATES**				
**AMC Presence**	**Absolute mortality difference (95% CI)**	**P-Value**	**Absolute mortality difference 95% CI**	**P-Value**
No	REFERENCE		REFERENCE	
Low	-0.2% (-0.3% to -0.03%)	0.02	-0.1% (-0.3% to 0.08%)	0.26
Moderate	-0.1% (-0.3% to 0.1%)	0.30	-0.08% (-0.3% to 0.2%)	0.56
High	-0.4% (-0.7% to -0.1%)	0.005	-0.5% (-0.9% to -0.1%)	0.01

*Linear probability model. Unadjusted model includes year, hospital random effects, region (Northeast, Midwest, West, South), principal discharge diagnosis. Model adjusting for patient characteristics further includes patient age, sex, Medicaid eligibility, and chronic conditions. Model incorporating market characteristics further includes Hospital Referral Region (HRR) population, median income, percentage of Black residents, physicians per 100,000 residents and hospital full-time equivalents per 1,000 population.

© 2023 Burke LG et al. *JAMA Network Open.*

eFigure 3. Variation in 30-Day Mortality Among Patients Hospitalized at Non-AMCs, Stratified by Market-Level AMC Presence, by Adjustment Model*

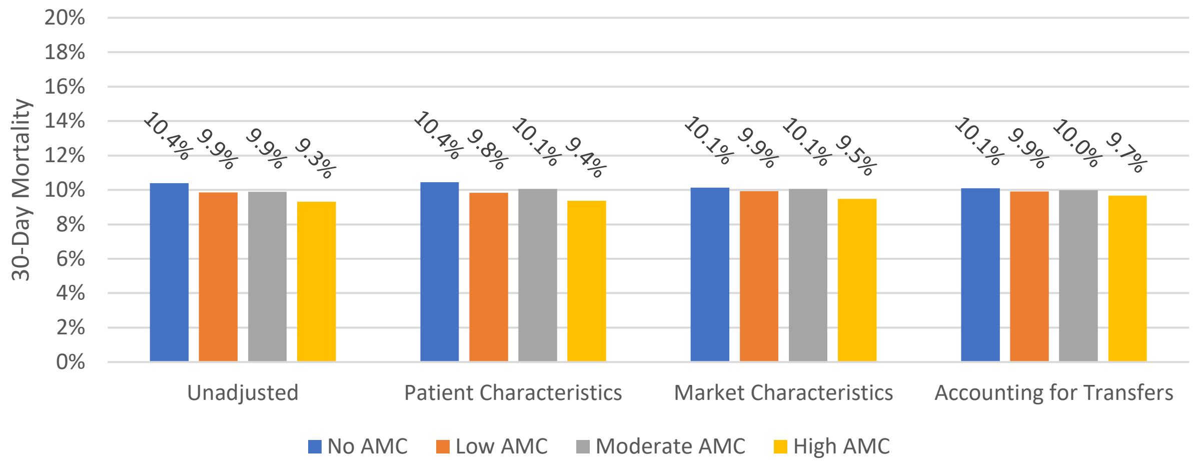

*Linear probability model with 30-day mortality as the outcome and market-level AMC presence as the predictor. Market category of AMC presence was defined as follows: no AMC presence (0% of hospitalizations managed at AMCs), low (>0 to 20%), moderate (>20% to 35%) and high AMC presence (>35%). The unadjusted model incorporates year, hospital random effects, geographic region, principal discharge diagnosis. The model incorporating patient characteristics further incorporates beneficiary age, sex, Medicaid eligibility and chronic conditions. The subsequent "market characteristics" model further incorporates the following Hospital Referral Region (HRR) characteristics: total population, percentage of Black residents, median income, physicians per 100,000 populations, hospital full time equivalent per 1000 populations and median income. The final model further incorporates the proportion of hospitalizations ending in transfer.

© 2023 Burke LG et al. *JAMA Network Open.*

eFigure 4. Variation in 90-Day Mortality Among Patients Hospitalized at Non-AMCs, Stratified by Market-Level AMC Presence, by Adjustment Model*

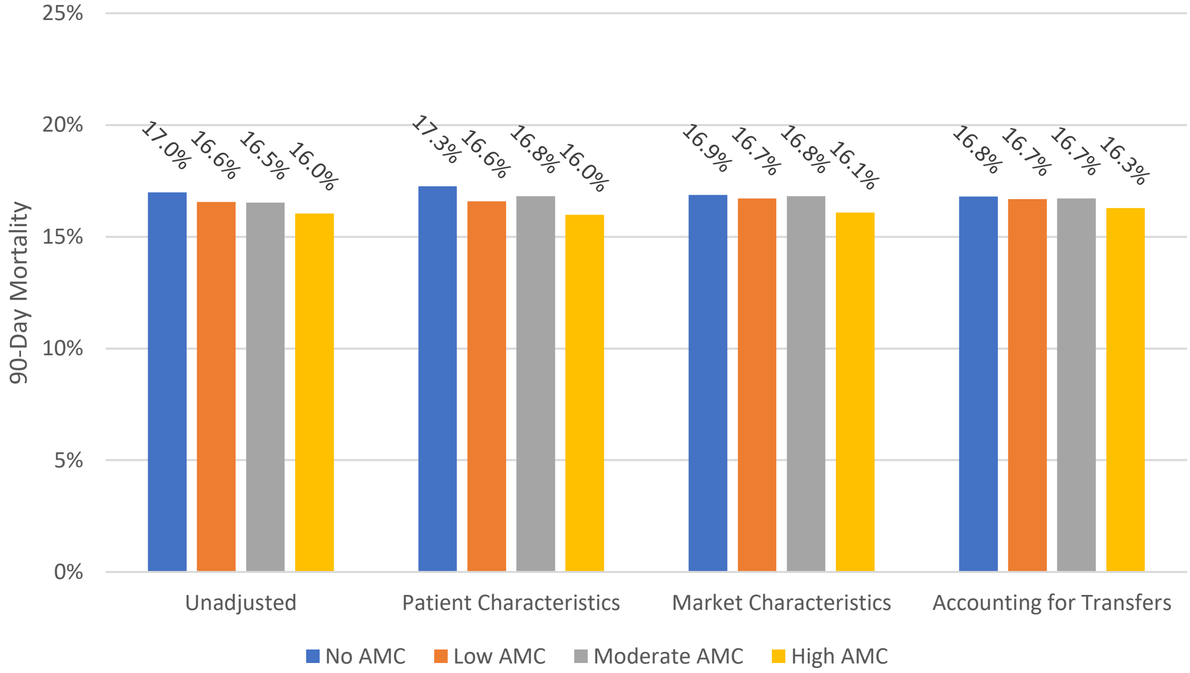

*Linear probability model. Market category of AMC presence was defined as follows: no AMC presence (0% of hospitalizations managed at AMCs), low (>0 to 20%), moderate (>20% to 35%) and high AMC presence (>35%). Unadjusted model includes year, hospital random effects, region (Northeast, Midwest, West, South), principal discharge diagnosis. Model adjusting for patient characteristics further includes patient age, sex, Medicaid eligibility, and chronic conditions. Model incorporating market characteristics further includes Hospital Referral Region (HRR) population, median income, percentage of Black residents, physicians per 100,000 residents and hospital full-time equivalents per 1,000 population. The final model further incorporates the proportion of hospitalizations ending in transfer.

© 2023 Burke LG et al. *JAMA Network Open.*

eTable 4. Comparison of 30- and 90-Day Healthy Days at Home (HDAH) at Non-Academic Medical Centers (Non-AMCs) by Category of Market-Level AMC Presence and Adjustment Model*

	30-Day HDAH		90-Day HDAH	
**UNADJUSTED**				
**AMC Presence**	**Difference (95% CI)**	**P-Value**	**Difference (95% CI)**	**P-Value**
No	REFERENCE		REFERENCE	
Low	-0.08 (-0.20 to 0.03)	0.16	-0.23 (-0.55 to 0.09)	0.15
Moderate	-0.17 (-0.34 to 0.01)	0.07	-0.11 (-0.60 to 0.38)	0.66
High AMC	-0.31 (-0.55 to -0.07)	0.01	-0.26 (-0.92 to 0.40)	0.43
**ADJUSTED FOR PATIENT CHARACTERISTICS**				
**AMC Presence**	**Difference (95% CI)**	**P-Value**	**Difference (95% CI)**	**P-Value**
No	REFERENCE		REFERENCE	
Low	0.44 (0.33 to 0.56)	<.001	1.00 (0.71 to 1.30)	<.001
Moderate	0.12 (-0.05 to 0.30)	0.17	0.57 (0.12 to 1.02)	0.01
High	0.51 (0.27 to 0.75)	<.001	1.74 (1.13 to 2.35)	<.001
**ADJUSTED FOR MARKET CHARACTERISTICS**				
**AMC Presence**	**Difference (95% CI)**	**P-Value**	**Difference (95% CI)**	**P-Value**
No	REFERENCE		REFERENCE	
Low	0.21 (0.07 to 0.34)	0.003	0.41 (0.07 to 0.76)	0.018
Moderate	0.003 (-0.18 to 0.19)	0.98	0.13 (-0.35 to .0.60)	0.60
High	0.38 (0.11 to 0.64)	0.005	1.25 (0.58 to 1.92)	<.001

*Linear regression model. Unadjusted model includes year, hospital random effects, region (Northeast, Midwest, West, South), principal discharge diagnosis. Model adjusting for patient characteristics further includes patient age, sex, Medicaid eligibility, and chronic conditions. Model incorporating market characteristics further includes Hospital Referral Region (HRR) population, median income, percentage of Black residents, physicians per 100,000 residents and hospital full-time equivalents per 1,000 population.

© 2023 Burke LG et al. *JAMA Network Open.*

eFigure 5. Variation in 30-Day Healthy Days at Home (HDAH) Among Patients Hospitalized at Non-AMCs, Stratified by Market-Level AMC Presence and Adjustment Model

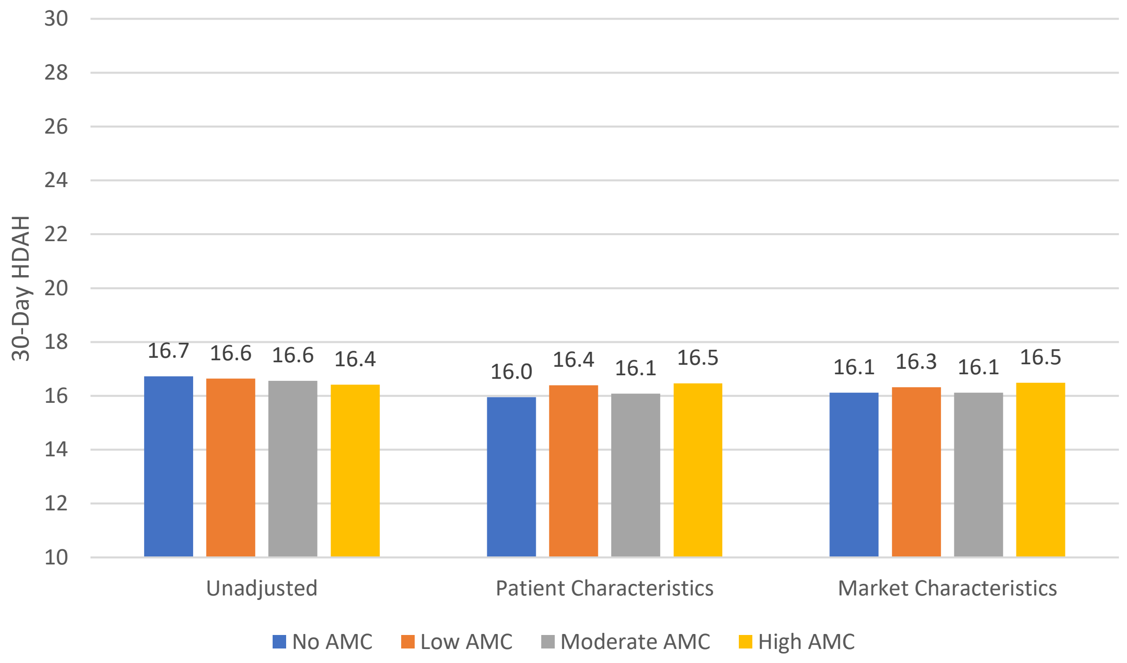

*Linear regression model. Unadjusted model includes year, hospital random effects, region (Northeast, Midwest, West, South), principal discharge diagnosis. Model adjusting for patient characteristics further includes patient age, sex, Medicaid eligibility, and chronic conditions. Model incorporating market characteristics further includes Hospital Referral Region (HRR) population, median income, percentage of Black residents, physicians per 100,000 residents and hospital full-time equivalents per 1,000 population.

© 2023 Burke LG et al. *JAMA Network Open.*

eFigure 6. Variation in 90-Day Healthy Days at Home (HDAH) Among Patients Hospitalized at Non-AMCs, Stratified by Market-Level AMC Presence and Adjustment Model*

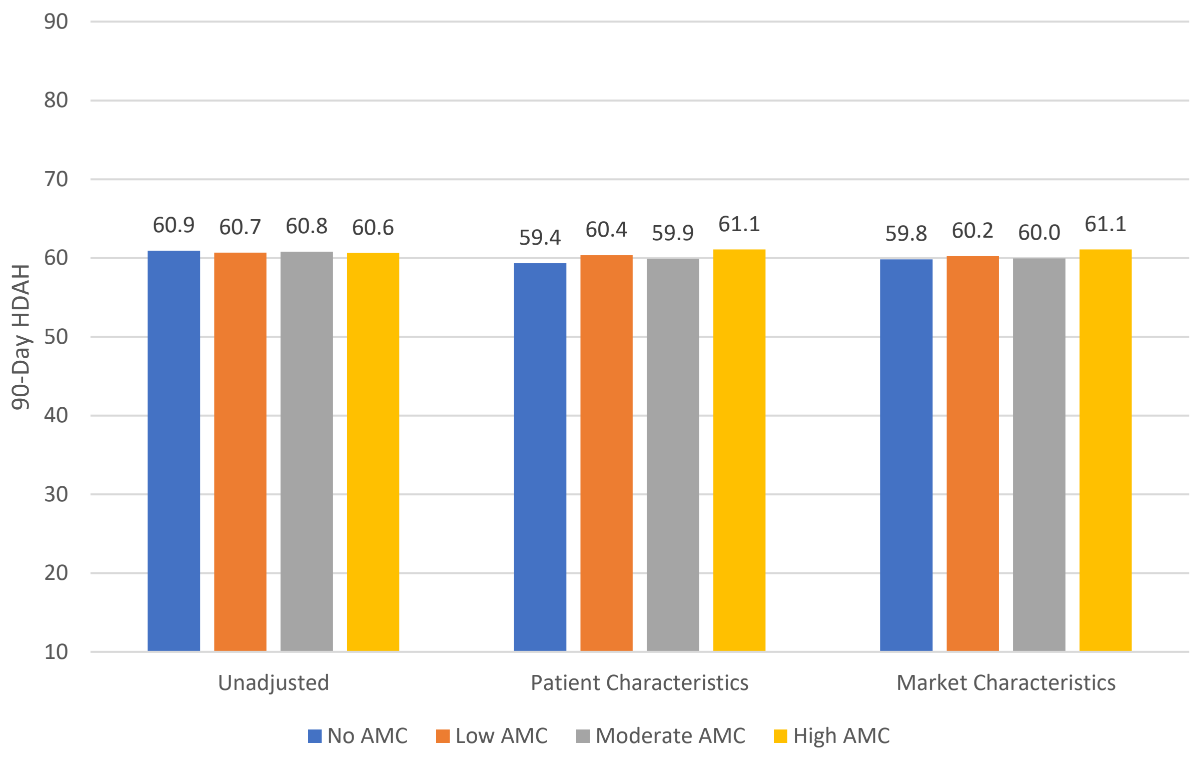

*Linear regression model. Unadjusted model includes year, hospital random effects, region (Northeast, Midwest, West, South), principal discharge diagnosis. Model adjusting for patient characteristics further includes patient age, sex, Medicaid eligibility, and chronic conditions. Model incorporating market characteristics further includes Hospital Referral Region (HRR) population, median income, percentage of Black residents, physicians per 100,000 residents and hospital full-time equivalents per 1,000 population.

© 2023 Burke LG et al. *JAMA Network Open.*

eFigure 7. Association Between Market-Level AMC Presence and Mortality for Patients Treated AMCs, Stratified by Adjustment Model

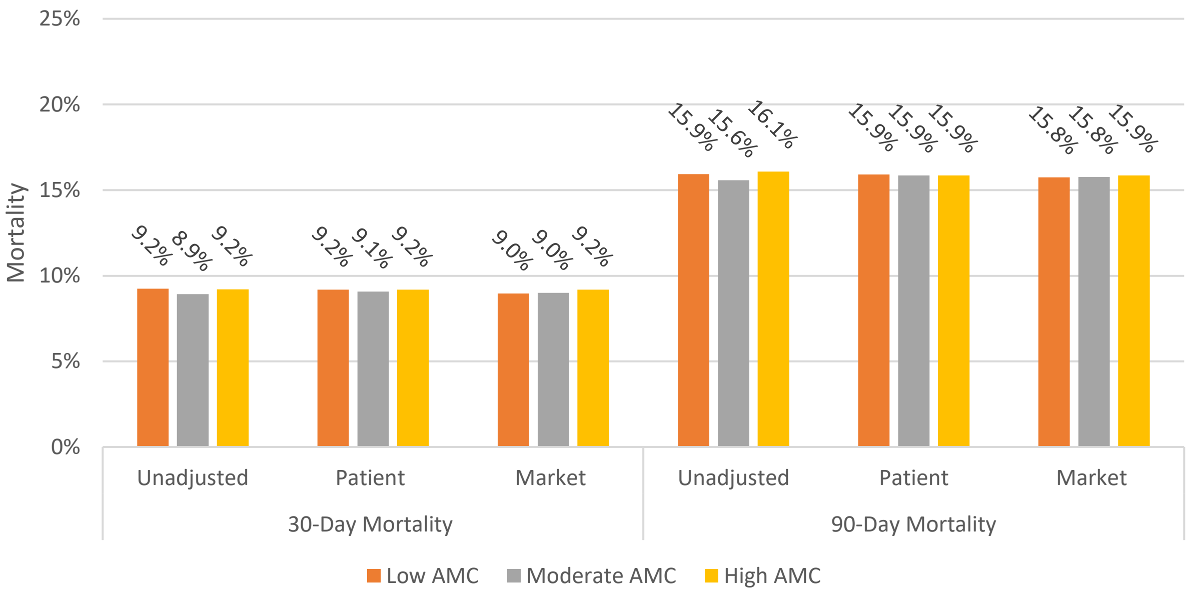

Linear probability model. Unadjusted model includes year, hospital random effects, region (Northeast, Midwest, West, South), principal discharge diagnosis. Model adjusting for patient characteristics further includes patient age, sex, Medicaid eligibility, and chronic conditions. Model incorporating market characteristics further includes Hospital Referral Region (HRR) population, median income, percentage of Black residents, physicians per 100,000 residents and hospital full-time equivalents per 1,000 population.

© 2023 Burke LG et al. *JAMA Network Open.*

eTable 5. Comparison of 30- and 90-Day Mortality at Academic Medical Centers (AMCs) by Category of Market-Level AMC Presence and Adjustment Model*

	30-Day Mortality		90-Day Mortality	
**UNADJUSTED**				
**AMC Presence**	**Absolute mortality difference (95% CI)**	**P-Value**	**Absolute mortality difference (95% CI)**	**P-Value**
Low	REFERENCE		REFERENCE	
Moderate	-0.31% (-0.73%, 0.10%)	0.14	-0.38% (-0.98%, 0.23%)	0.22
High AMC	-0.04% (-0.47%, 0.40%)	0.87	0.15% (-0.49%, 0.79%)	0.64
**ADJUSTED FOR PATIENT CHARACTERISTICS**				
**AMC Presence**	**Absolute mortality difference (95% CI)**	**P-Value**	**Absolute mortality difference (95% CI)**	**P-Value**
Low	REFERENCE		REFERENCE	
Moderate	-0.10% (-0.49%, 0.30%)	0.63	-0.07% (-0.60%, 0.45%)	0.78
High	-0.06% (-0.48%, 0.35%)	0.77	-0.07% (-0.63%, 0.48%)	0.80
**ADJUSTED FOR MARKET CHARACTERISTICS**				
**AMC Presence**	**Absolute mortality difference (95% CI)**	**P-Value**	**Absolute mortality difference (95% CI)**	**P-Value**
Low	REFERENCE		REFERENCE	
Moderate	0.03% (-0.4%, 0.4%)	0.89	0.01% (-0.53%, 0.55%)	0.98
High	0.23% (-0.24%, 0.70%)	0.34	0.10% (-0.53%, 0.73%)	0.76

*Linear probability model. Unadjusted model includes year, hospital random effects, region (Northeast, Midwest, West, South), principal discharge diagnosis. Model adjusting for patient characteristics further includes patient age, sex, Medicaid eligibility, and chronic conditions. Model incorporating market characteristics further includes Hospital Referral Region (HRR) population, median income, percentage of Black residents, physicians per 100,000 residents and hospital full-time equivalents per 1,000 population.

© 2023 Burke LG et al. *JAMA Network Open.*

eFigure 8. A LOESS (Locally Estimated Scatterplot Smoothing) Plot of the Relationship Between Unadjusted 30-Day Mortality for Patients Treated at Non-AMCs and HRR-Level AMC Presence*

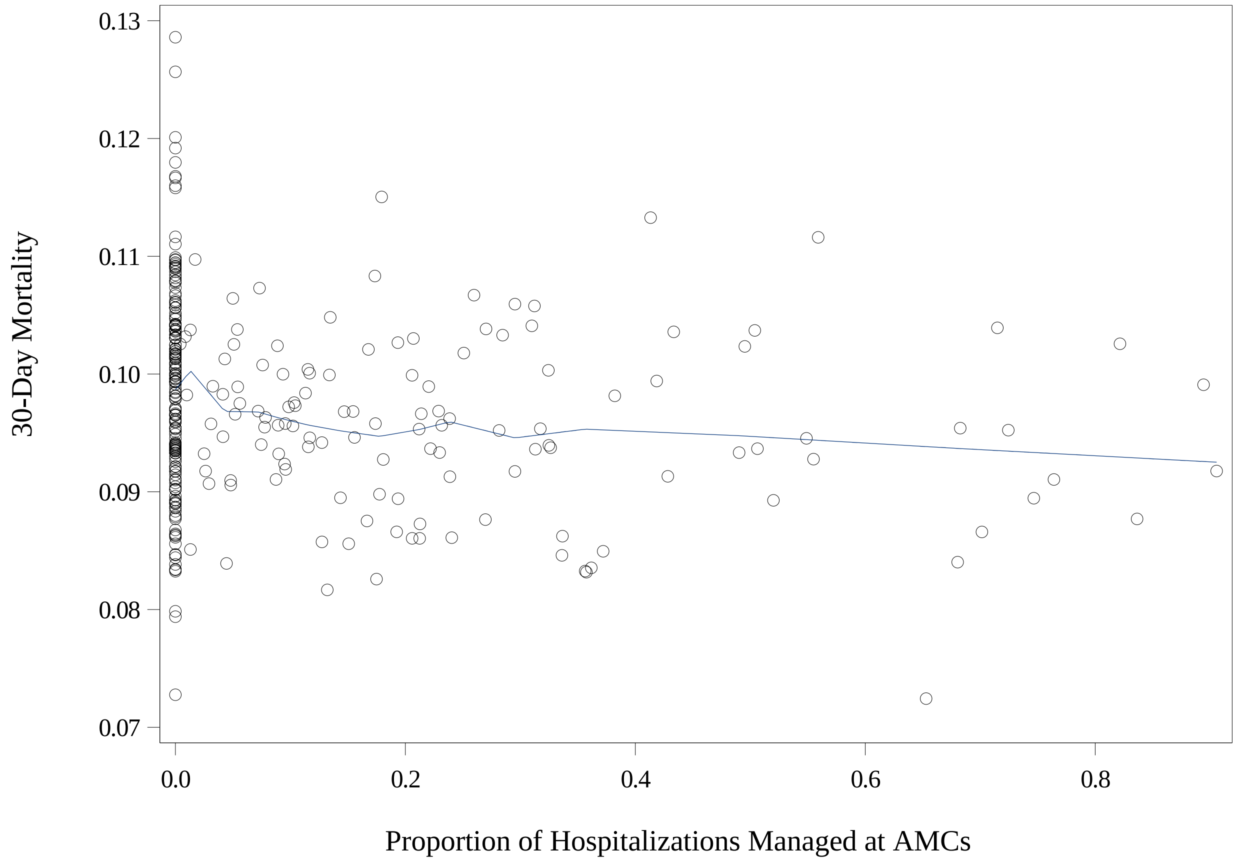

© 2023 Burke LG et al. *JAMA Network Open.*
