# Supplementary material for: Association of Academic Medical Center Presence With Clinical Outcomes at Neighboring Community Hospitals Among Medicare Beneficiaries
Source: JAMA Netw Open. 2023 Feb 1;6(2):e2254559. doi: 10.1001/jamanetworkopen.2022.54559 (PMC9892959; doi:10.1001/jamanetworkopen.2022.54559)
Supplement: Supplement 2. — Data Sharing Statement [file jamanetwopen-e2254559-s002.pdf]

Data Sharing Statement

Burke. Association of Academic Medical Center Presence With Clinical Outcomes at Neighboring Community Hospitals Among Medicare Beneficiaries. *JAMA Netw Open*. Published February 01, 2023. doi:10.1001/jamanetworkopen.2022.54559

Data
Data available: No

Additional Information
Explanation for why data not available: Our study is observational in nature and governed by a Data Use Agreement with the Centers for Medicare and Medicaid Services which limits sharing of the data, which contains protected Health Information.
